# The IS*6* family, a clinically important group of insertion sequences including IS*26*

**DOI:** 10.1186/s13100-021-00239-x

**Published:** 2021-03-23

**Authors:** Alessandro Varani, Susu He, Patricia Siguier, Karen Ross, Michael Chandler

**Affiliations:** 1 School of Agricultural and Veterinary Sciences, Universidade Estadual Paulista, Jaboticabal, Sao Paulo Brazil; 2 State Key Laboratory of Pharmaceutical Biotechnology, Medical School of Nanjing University, Nanjing, 210093 Jiangsu China; 3 Centre de Biologie Intégrative-Université Paul SABATIER, CNRS - Laboratoire de Microbiologie et Génétique Moléculaires, UMR 5100 - bât. CNRS-IBCG, Toulouse, France; 4 Protein Information Resource, Department of Biochem., Mol. and Cell. Biol, Georgetown University Medical Center, Washington, DC USA; 5 Department of Biochem., Mol. and Cell. Biol, Georgetown University Medical Center, Washington, DC USA

**Keywords:** Insertion sequence, Phylogeny, Genome impact, Transposition mechanisms, Clinical importance, Antibiotic resistance

## Abstract

The IS*6* family of bacterial and archaeal insertion sequences, first identified in the early 1980s, has proved to be instrumental in the rearrangement and spread of multiple antibiotic resistance. Two IS, IS*26* (found in many enterobacterial clinical isolates as components of both chromosome and plasmids) and IS*257* (identified in the plasmids and chromosomes of gram-positive bacteria), have received particular attention for their clinical impact. Although few biochemical data are available concerning the transposition mechanism of these elements, genetic studies have provided some interesting observations suggesting that members of the family might transpose using an unexpected mechanism. In this review, we present an overview of the family, the distribution and phylogenetic relationships of its members, their impact on their host genomes and analyse available data concerning the particular transposition pathways they may use. We also provide a mechanistic model that explains the recent observations on one of the IS*6* family transposition pathways: targeted cointegrate formation between replicons.

**Supplementary Information:**

The online version contains supplementary material available at 10.1186/s13100-021-00239-x.

## Introduction

The importance of insertion sequences (IS) in shaping prokaryotic genomes and in directing gene sequestration as a prologue to horizontal transfer in bacterial populations has been well documented (see [1, 2] and references therein for a detailed discussion). IS are small DNA segments generally less than 2.5 kb long encoding an enzyme, the transposase (Tnp), which catalyzes the DNA cleavage and strand-transfer reactions enabling movement from one location (the donor site) to another (the target site) in DNA molecules. Tnp acts on its cognate IS and is generally the only gene carried by the IS. IS are a diverse group of transposable elements (TE) which often include short imperfect terminal inverted repeat sequences (IR) and generate small flanking directly repeated target DNA sequences (DR) on insertion. There are at least 27 IS families [3, 4] defined by the chemistry used by their Tnp, the sequence relatedness of the Tnp as defined [5] by TRIBE-MCL which relies on the Markov cluster (MCL) algorithm [6] and the sequence of their ends. These families are listed in the ISfinder database (ISfinder, https://www-is.biotoul.fr/) and described in detail in TnPedia (https://tnpedia.fcav.unesp.br/index.php/Main_Page), a source of information on prokaryotic TE which is integrated into the transposon database, TnCentral (https://tncentral.proteininformationresource.org/).

Here we present an overview of one of these IS families, IS*6*, whose importance in generating clusters of clinically important antibiotic resistance genes is becoming increasingly clear [7] and whose members may use an unusual transposition pathway.

### IS*6* nomenclature and initial identification

**Fig. 1:**
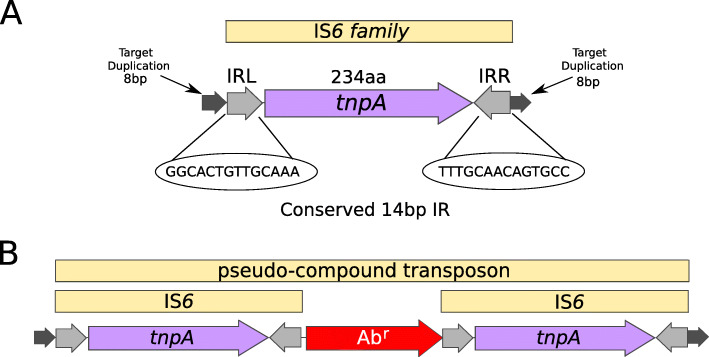
IS*6* family organization. **Top**. Structure of IS*6* family. The IS is represented by a yellow bar. Left (IRL) and right (IRR) terminal 14 bp IRs are shown as grey filled arrows with the DNA sequence below. The 8 bp direct target repeats are shown as black filled arrows. The transposase open reading frame is shown in purple and its orientation is indicated by the arrow head. **Bottom.** A Pseudo-compound transposon (see text for explanation). IS6 family characteristics are as above. A generic antibiotic resistance gene AB^r^ is shown in red

**Fig. 2:**
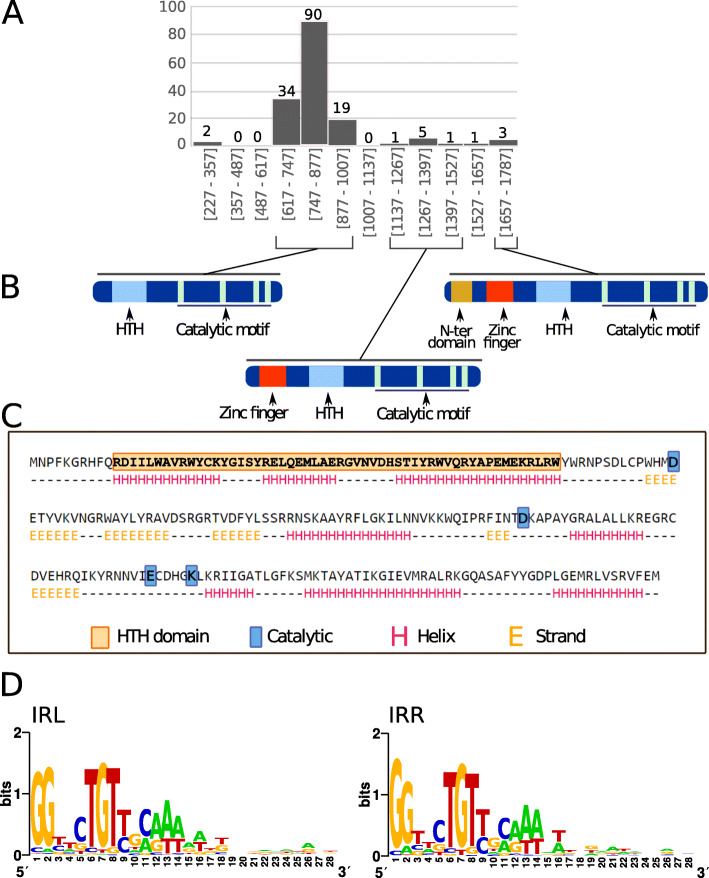
The general characteristics of the IS*6* family. **a**: Distribution of IS length (base pairs). The number of examples used in the sample is shown above each column. **b:** shows the domain structure of IS6 family transposases with a helix-turn-helix domain (HTH) and a catalytic domain with the Characteristic DDE triad followed by a K/R residue, and, in the case of the middle section, an additional zinc finger motif present in the longer members of the family (clade h) while in the righthand section an additional N-terminal domain is present (clade i). **c:** Secondary structure prediction of TnpA IS*26* by Jpred [24]. **d**: Left (IRL) and right IRR inverted terminal repeats are shown in WebLogo format [25]

There are at present (December 2020) nearly 160 IS*6* family members in ISfinder (https://www-is.biotoul.fr/scripts/search-db.php) from nearly 80 bacterial and archaeal species, although this represents only a fraction of those present in the public databases. The family was named [8] after the directly repeated insertion sequences in transposon Tn*6* [9] to standardize the various names that had been attributed to identical elements (e.g. IS*15*, IS*26*, IS*46*, IS*140,* IS*160*, IS*176*) [10–22] including one isolate, IS*15*, corresponding to an insertion of one iso-IS*6* (IS*15*Δ) into another [11]. More recently, there has been some attempt to rename the family as the IS*26* family (see [23]) because of accumulating experimental data from IS*26* itself, and the importance of this IS in accumulation and transmission of multiple antibiotic resistance, although this might potentially introduce confusion in the literature. IS*6* family members have a simple organization (Fig. 1) and generate 8 bp direct target repeats on insertion. This family is very homogenous with an average length of about 800 bp for the majority (between 700 and 890 bp) and highly conserved short, generally perfect, IRs (Fig. 2a). There are two examples of MITES (**M**iniature **I**nverted repeat **T**ransposable **E**lements composed of both IS ends and no intervening orfs [26]; of 227 and 336 bp), 7 members between 1230 and 1460 bp and three members between 1710 and 1760 bp. One member, IS*15*, of 1648 bp represents and insertion of one IS into another [10, 12]. Many members are found as part of compound transposons (called pseudo-compound transposons [8] (Fig. 1) described below [23]) invariably as flanking *direct repeats* (Fig. 1), a consequence of their transposition mechanism [14, 16, 20, 21, 27–39].

### Distribution and phylogenetic Transposase tree

**Fig. 3:**
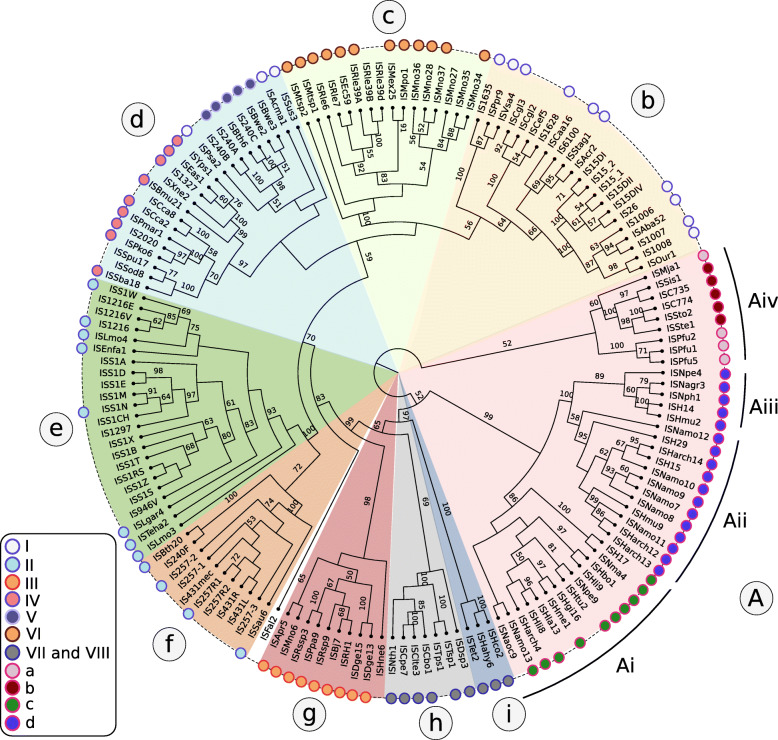
A dendrogram of IS6 family members.: A dendrogram of IS6 family members. The figure shows 11 major clades. The surrounding colored circles and the insert indicate the clades identified by [40]. The insert shows the correspondence between the clades from Harmer and Hall and those defined here. **Clades: A:** composed almost entirely of archea; **Ai**: (*n* = 12) is composed of diverse *Halobacterial species* (*Halohasta, Haloferax, Natrinema, Natrialba, Halogeometricum, Natronomonas, Natronococcus,* and *Haloarcula*); **Aii:** (n = 12) is composed uniquely of *Halobacterial Euryarchaeota*; **Aiii:** (*n* = 5) is composed entirely of *Halobacterial Euryarchaeota* (*Haloarcula, Halomicrobium, Natronomonas, Natronobacterium, Natrinema*); **Aiv:** (*n* = 9) which includes both *Euryarchaeota* and *Crenarchaeota;* **b:** (*n* = 16) *Actinobacteria*, *Alpha*-, *Beta*-, and *Gamma-proteobacteria;* **c:** (*n* = 14) *Alphaproteobacteria*: *Rhizobiaceae* and *Methylobacteriaceae*); **d:** (*n* = 24) (Alpha-, Beta-, and Gamma-*proteobacteria*, *Firmicutes*, *Cyanobacteria*, *Acidobacteria* and Bacteroidetes); **e:** (*n* = 23) is composed mainly of IS from Lactococcus, a single *Leuconostoc* and other bacilli (Lysteria, Enterococcus); **f:** (*n* = 11) largely *Staphylococci* with 2 *B. thuringiensis*; **g:** (*n* = 10) is heterogenous (*Alpha proteobacteria: Methylobacterium, Paracoccus, Roseovarius, Rhizobium, Bradyrhizobium; Deinococci* and *Halobacteria*); **h:** (n = 5) composed entirely of *Firmicutes* (*Natranaerobius*, *Clostridium* and *Thermoanaerobacter*); **i:** (*n* = 3) is composed of Halanaerobia and Thermoanaerobacter. TnpA protein sequences retrieved from ISfinder curated data set were aligned with MAFFT 7.309, and their best-fit evolutionary models were predicted with ProTest 3.2.4. A maximum likelihood tree was reconstructed with RaxML 8.2.9 using a bootstrap value of 1000. The final tree was visualized in FigTree 1.4.4 (http://tree.bio.ed.ac.uk/software/figtree) and edited with Inkscape 0.92.4 (http://www.inkscape.org)

A phylogenetic tree based on the transposase amino acid sequence of the ISfinder collection (Fig. 3) shows that the IS*6* family members fall into a number of well-defined clades. This slightly more extensive set of IS corresponds well to the results of another wide-ranging phylogenetic analysis [40]. These clades include one which groups all archaeal IS*6* family members composed mainly of *Euryarchaeota* (*Halobacteria*; Fig. 3 Ai-iii). Group Aiv includes both *Euryarchaeota* (*Thermococcales* and *Methanococcales*) and *Crenarchaeota* (*Sulfolobales*). Of the nine clades containing bacterial IS: clade b includes some Actinobacteria, Alpha-, Beta-, and *Gamma-proteobacteria*; clade c is more homogenous and is composed of *Alphaproteobacteria* (*Rhizobiaceae* and *Methylobacteriaceae*); clade d includes examples from the Alpha-, Beta-, and Gamma-*proteobacteria*, *Firmicutes*, *Cyanobacteria*, *Acidobacteriia* and Bacteroidetes; clades e and f are composed exclusively of Firmicutes (almost exclusively *Lactococci* in the case of clade e); clades g and h are more mixed and clade I contains only three examples. As might be expected, transposase length is approximately correlated with the clades. For example, family members from the archaea tend to be slightly smaller, in the range of 700–750 for clades Ai and Aii while members of clades h and i all carry the longest transposase genes (1230 to 1460 bp and 1710 to 1760 bp respectively). The division into clades is also underlined to some extent by the IR sequences where the sequence motifs are more pronounced when each clade is considered separately (Fig. S1) (see “Organization” below).

Clearly, the ISfinder collection does not necessarily accurately reflect the IS*6* family distribution and these groupings should be interpreted with care. For example, although many are not included in the ISfinder database, IS*6* family elements are abundant in archaea and cover almost all of the traditionally recognized archaeal lineages (methanogens, halophiles, thermoacidophiles, and hyperthermophiles [41] (Fig. 3).

MCL analysis [6] for the entire group of transposases using the criteria of ISfinder for classification [5] showed that all members fell within the definition of a single family (Inflation factor 1.2, score > 30) and fell into 3 groups: clades b-I; clades Ai-Aiii; and clade Aiv using the appropriate filter (Inflation factor 2, score > 140). The answer to the recent question “An analysis of the IS*6*/IS*26* family of insertion sequences: is it a single family?” [40] is therefore, “Probably, yes” according to the ISfinder definition.

A recent study [42] identified a number of IS*26* variants with specific mutations in their Tpases. In particular one variant, originally called IS*15Δ* [11, 43] was observed to exhibit enhanced activity and it was suggested that such mutants, even though they satisfy ISfinder criteria attributing a new name for an IS (< 95% nucleotide identity and/or < 98% amino acid identity). It has been suggested that such variant should be suffixed as IS*26*.v1, .v2 etc. [42]. This makes sense if the mutation is not functionally neutral and results in a change IS properties or behavior.

#### Finding new family members: the strategy

As for all insertion sequences, there are no fully automated methods for identification of IS*6* family members in the public databases and ISfinder has not undertaken an extensive database survey. As a general strategy, potential IS sequences can be compared to those in the ISfinder database using the online BLAST tool (https://www-is.biotoul.fr/blast.php) or TnCentral (https://tncentral.proteininformationresource.org/tn_blast.html). For genome or plasmid analysis the semiautomatic genome annotation tool (Varani et al. 2011) (ISsaga: http://issaga.biotoul.fr/issaga_index.php) can be used. This is integrated into ISfinder and compares sequences with those in the ISfinder database. There are number of other software tools available, showing different funcionalities, sensivity and precision that can be used for *ab initio* IS identification. We highlight some of these, such as OASIS [44], ISEScan [45],and ISQuest [46]. However, no software performs better than the classic and manual annotation based on strucutural features and genetic characterisitiscs present in each IS family. In fact, there are still many bioinformatic challenges in obtaining a complete and proper IS identification in a given bacterial genome. For instance, it is important to ascertain manually whether the potential IS includes both terminal inverted repeats (IR; see **Organization: Terminal Inverted Repeats** below) and whether or not the IS is flanked by direct target repeats (DR). This will indicate whether the IS is functional (complete IR) and how it may have moved to its present position (presence or absence of DR; see **Mechanism: the state of play** below**).**

### Genomic impact and clinical importance

Activity resulting in horizontal dissemination is suggested, for example, by the observation that copies identical to IS*6100* originally identified in *Mycobacterium fortuitum* [47](Fig. 3, clade b) occur in other bacteria: as part of a plasmid-associated catabolic transposon carrying genes for nylon degradation in *Arthrobacter sp.* [48]; in the *Pseudomonas aeruginosa* plasmid R1003 [49]; and in integrons of the In4-type from transposons such as Tn*1696* [50, 51] and *Xanthomonas campestris* transposon Tn*5393b* [52]. Similar copies have also been reported in *Salmonella enterica* (typhimurium) [53], and on plasmid pACM1 from *Klebsiella oxytoca* (AF107205) [54].

#### Passenger genes

A number of IS families contain members, called tIS which carry passenger genes. A single member of the family, IS*Dsp3*, present in a single copy in *Dehalococcoides sp.* BAV1 carries a passenger gene annotated as a hypothetical protein.

#### Expression of neighboring genes

The formation of hybrid promoters on insertion, where the inserted element provides a − 35 promoter component and the flanking sequence carries a − 10 promoter component, is clearly a general property of members of the IS*6* family [31, 55–59] and occurs frequently.

IS*257* [60] (Fig. 3, clade f) (also known as IS*431*), which plays an important role in sequestering a variety of antibiotic resistance genes in clinical isolates of methicillin-resistant *Staphylococcus aureus* (MRSA) (e.g. [7, 55, 56, 61, 62], provides an outward-oriented promoter which drives expression of genes located proximal to the left end. Moreover, both left and right ends appear to carry a − 35 promoter component which would permit the formation of hybrid promoters on insertion of the IS next to a resident − 10 element [56, 62, 63]. Insertion can result in activation of a neighboring gene using both a hybrid promoter and an indigenous promoter [56]. IS*257* is also involved in expression of *tetA* [63] and *dfrA* [55] in *S. aureus.* This is also true of IS*26* which forms hybrid promoters shown to drive antibiotic resistance genes such as *aphA7* (*Pasteurella piscicida* [64] *Klebsiella pneumoniae* [31])*, bla*_*SHV-2a*_ (*Pseudomonas aeruginosa* [65]) and wide spectrum beta-lactam resistance gene *bla*_KPC_ [2, 4]. While IS*6100* [47] (Fig. 3, clade b)*,* often used as an aid in classifying mycobacterial isolates [66–68], drives *strA strB* expression in *X. campestris* pv. vesicatoria [52].

#### Pseudo-compound transposons

This IS family is able to form transposons which resemble compound transposons with the flanking IS in direct repeat but, because of the particular transposition mechanism of IS*6* family members which involves the formation of cointegrates (see below), these were called pseudo-compound transposons [8, 23]. They include Tn*610* (flanked by IS*6100* [47]), Tn*4003* and others (flanked by IS*257* [7, 61, 69]), Tn*2680* [13] and Tn*6023* (flanked by IS*26* [70]).

### IS*26* and the clinical landscape

In view of the particular importance of IS*26* in clinical settings it is worthwhile devoting a separate section to the contribution of this IS to the clinical landscape. IS*26* [13–15](Fig. 3, clade b) is encountered with increasing frequency in plasmids of clinical importance where it is involved in: sequestering antibiotic resistance genes and generating arrays of these genes in clinically important conjugative plasmids and in the host chromosome; expression of antibiotic resistance genes; and other plasmid rearrangements (see [7, 37, 71–76]).

Recognition of its place as an important player has derived from the large number of sequences now available of multiple antibiotic resistance plasmids and chromosomal segments such as Genomic Resistance Islands (GRI). It is now no longer practical to provide a complete analysis of the literature (A PubMed search (19th November 2020) using IS*26* as the search term yielded nearly 450 citations). The references in the following are not exhaustive but simply provide examples.

#### IS*26* arrays

**Fig. 4:**
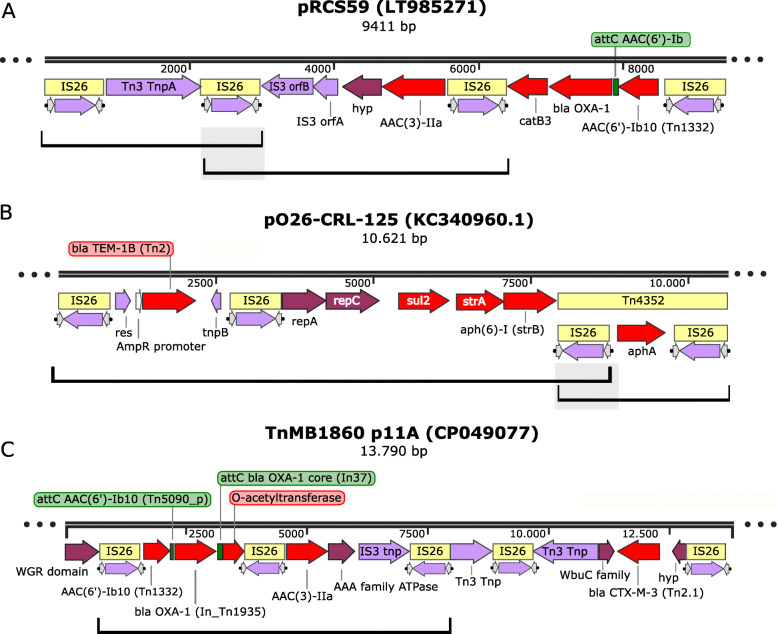
IS26 arrays. Genbank accession numbers for the DNA segments are shown in parentheses. Images were initially created using SnapGene. Open reading frames are shown as horizontal boxes where the arrowheads indicate the direction of translation. Red, antibiotic resistance genes; lavender, transposase related genes; purple, other; yellow boxes, IS copies; green boxes, integron cassette recombination sites; the terminal IRs are also shown. Grey boxes show the overlap between potential transposons. The figure shows **a**) overlapping potential transposons from plasmid pRCS59. **b**) plasmid pO26-CRL-125. **c**) the TnMB1860 DNA segment (Shropshire et al., 2020) PMID: 33164081). The major amplified segment is indicated by a horizontal bracket below. The horizontal brackets in **a**) and **b**) indicate overlapping potential transposons. The horizontal bracket in **c**)

IS*6* family members are often found in arrays (Fig. 4) in direct and inverted repeat in multiple drug resistant plasmids (e.g. *Salmonella typhimurium* [37, 70, 77], *Klebsiella quasipneumoniae* [78], *Acinetobacter baumannii* [74, 79], *Proteus mirabilis* [80] and uncultured sewage bacteria [81] among many others). These are often intercalated in or next to other transposable elements rather than neatly flanking antibiotic resistance genes and can form units able to undergo tandem amplification.

#### IS26-mediated gene amplification

Early studies with Tn*1525* (from *Salmonella enterica* serovar Panama), in which an *aphA1* (*aph* (3′) (5″)-I) gene is flanked by two directly repeated copies of the IS*6* family member, IS*15*, reported tandem amplification of *aphA1* when the host was challenged by kanamycin [82]. Restriction enzyme mapping was used to demonstrate that the amplified segments were of the type IS-aph-IS-aph-IS-aph-IS but no direct sequence data is available. Amplification was thought to occur by homologous recombination between two flanking IS*15* copies since it occurred in a wildtype host but the transposon was stable in a *recA*^−^ genetic background. Another example was observed in clinical isolates of *Acinetobacter baumannii* following continuous antibiotic treatment treatment of a single patient with Tobramycin over a period of days*.* Amplification occurred with Tn6*020*, an IS*26*-based transposon in which the flanking IS bracket a similar *aphA1* gene and could also be reproduced in bacterial culture [83]. It should be noted that the left hand IS*26* includes an additional abutting 175 bp IS*26* fragment (partial sequence JF343535). In this case, the amplified unit was proposed to be IS-*aph*-IS-IS-*aph*-IS-IS-*aph*-IS. This structure would clearly be unusual but may be due to a misinterpretation of the depth of coverage of the region or to the unusual structure of the transposon. In addition, the amplified transposon had inserted into a known target prior to amplification generating the expected eight base pair target repeat but an 8 bp segment between the first DR and the first IS end (DR-8 bp-IS-*aph*-IS-IS-*aph*-IS-IS-*aph*-IS … DR). A third example [84] was identified during a study of clinical isolates of non-carbapenemase-producing Carbapenem-Resistant Enterobacteria, non-CP-CRE, isolated from several patients with recurrent bacteraemia. An increase in carbapenem resistance occurred partially due to IS*26*-mediated amplification up to 10 fold of a DNA segment carrying *bla*_OXA-1_ and *bla*_CTX-M-1_ genes. These form part of a larger chromosomal structure of IS*26* arrays which they call TnMB1860 (Fig. 4). It was unclear whether this cassette amplification was due to transposition activity or, gene amplifications such as those observed with IS*1* [85–90] which may occur by replication slippage between direct repeats or by unequal crossing-over [91, 92].

Another example has been revealed by Hastak et al. [93] who analysed a multi resistant derivative of the clinically important, globally dispersed pathogenic, *Escherichia coli* ST131 subclade H30Rx, isolated from a number of bacteraemic patients and revealed that increased piperacillin/tazobactam resistance was due to IS*26*-mediated amplification of *bla*_TEM-1B_. A similar type of limited (tandem dimer) amplification of an IS*26*-flanked *bla*_SHV-5_-carrying DNA segment found in plasmids from a number of geographically diverse enteric species was identified in a nosocomial *Enterobacter cloacae* strain [94]. A more extensive amplification (> 10 fold) was observed with the same DNA segment located in a different plasmid in a well-characterised laboratory strain of *Escherichia coli* and occurred in a *recA*-independent manner [72]. While even higher levels of tandem amplification (~ 65 fold) of the *aphA1* gene were identified in the IS*26*-based Tn*6020* in *Acinetobacter baumannii* [83].

#### IS*26*-mediated plasmid Cointegration

The earliest studies on this family of IS demonstrated that they could generate cointegrates as part of the transposition mechanism (see **Cointegrate formation** below) [12, 14, 16, 19, 20, 39].

Several studies have now demonstrated that this can occur in a clinical setting. For example, plasmid pBK32533 (KP345882) [95], carried by *E. coli* BK32533 isolated from a patient with a urinary tract infection is an IS*26*-mediated cointegrate between *Klebsiella pneumoniae* BK30661 plasmid pBK30661 (KF954759) [96] and a relative of *Salmonella enterica* p1643_10 (KF056330) [97]. Interestingly, the flanks of the IS*26* copies at the junction of the two plasmids are TGTTTTTT-IS-TTATTAAT and TTATTAAT-IS-TGTTTTTT. The most parsimonious explanation would be that pBK32533 was generated in a multi-step inter-molecular transposition event: in one step, an IS*26* copy from an unknown source used a TTATTAAT target sequence in pBK30661 and this cointegrate was then resolved resulting in pBK30661 containing an IS26 copy flanked by the target repeat (TTATTAAT-IS*26*-TTATTAAT) and, in a second step, a TGTTTTTT sequence in p1643_10 was targeted by the pBK30661 IS*26* to generate the final cointegrate in which the two IS*26* copies are flanked by the observed target sequences. Additional examples have been identified in KPC-producing *Proteus mirabilis* [80] and in *Klebsiella pneumoniae* also involving inversions [76, 98].

### Organization

IS*6* family members range in length from 789 bp (IS*257*) to 880 bp (IS*6100*) (Fig. 2a) and generally create 8 bp direct flanking target repeats (DR) on insertion [13]..

### The transposase

A single transposase *orf* is transcribed from a promoter at the left end and stretches across almost the entire IS. The putative transposases (Tpases) are between 213 (IS*15*) and 254 (IS*6100*) amino acids long with a majority in the 220–250 amino acid range. They are very closely related and show identity levels ranging from 40 to 94% with a helix-turn-helix (HTH) and a typical catalytic motif (DDE) (Fig. 2b, c, Fig. S2). Translation products of this frame have been demonstrated for IS*240* [35]. However, the 7 members of clade h, all from *Clostridia*, are somewhat larger (340–350 amino acids) as a consequence of an N-terminal extension in the transposases with a predicted Zinc Finger (ZF) composed of several CxxC motifs (Fig. 2b; Fig. S2). A Blast analysis of the non-redundant protein database at NCBI revealed a large number of IS*6* family transposases of this type (data not shown). The vast majority of these were from Clostridial species. In addition, the transposases of members of clade i (450 amino acids) have both the ZF domain and a supplementary N-terminal extension (Fig. 2b).

Several members (e.g. IS*Rle39a*, IS*Rle39b* and IS*Enfa1*) apparently require a frameshift for Tpase expression. It is at present unclear whether this is biologically relevant. However, alignment with similar sequences in the public databases suggests that IS*Enfa1* itself has an insertion of 10 nucleotides and is therefore unlikely to be active.

### Transposase expression

In the case of IS*26*, the promoter is located within the first 82 bp of the left end and the intact *orf* is required for transposition activity [15], Little is known concerning the control of transposase expression although transposition activity of IS*6100* in *Streptomyces lividans* [99] is significantly increased when the element is placed downstream from a strong promoter. This is surprising since IS generally incorporate mechanisms to restrict transposition induced by insertion into highly transcribed genes (e.g. [100] and references therein).

### Terminal inverted repeats

All carry short related (15–20 bp) terminal IR. As shown in Fig. 2d, in spite of the wide range of bacterial and archaeal species in which family members are found, there is a surprising sequence conservation. In particular, the presence of a G dinucleotide at the IS tips and cTGTt and caaa internal motifs (where uppercase letters are fully conserved and lowercase letters are strongly conserved nucleotides). Sequence motifs are more pronounced when each clade is considered separately (Fig. S1).

### Mechanism: the state of play

Early studies suggested that IS*6* family members give rise exclusively to replicon fusions (cointegrates) in which the donor and target replicons are separated by two directly repeated IS copies (e.g. IS*15D*, IS*26*, IS*257*, IS*1936*) [12, 14, 16, 20, 101]. More recent results principally with IS*26* have suggested that, perhaps like IS*1* (IS*1* family) [102] and IS*903* (IS*5* family) [103, 104], members of this IS family may be able to transpose using alternative pathways [23, 105–107].

### Cointegrate formation

**Fig. 5:**
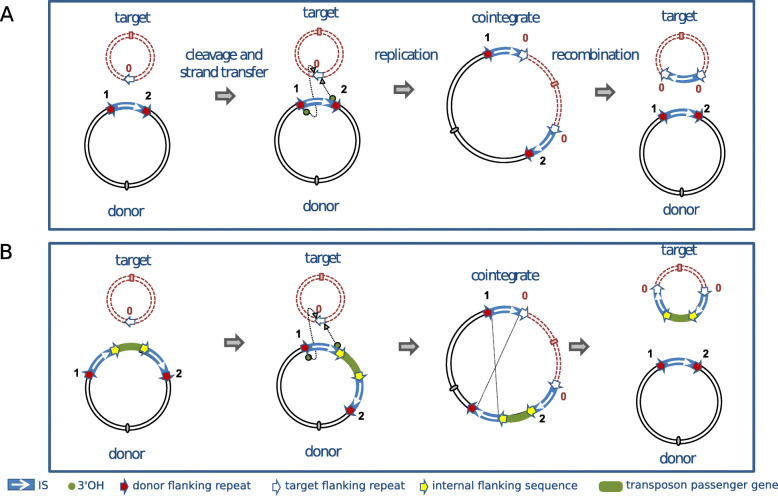
Intermolecular transposition models. **a:** classical replicative cointegration [108]. Modified from [98]. Donor DNA is shown in black, target DNA as a red dotted line. Replication origins on each molecule are represented by a small oval. The IS is shown as a blue box with the white arrow indicating the direction of expression of the transposase. The small directly repeated flanking sequences generated by insertion are shown as red arrows. The target sequence destined to become the new flanking repeat is indicated by white arrows. Transposition is initiated by cleavage at both terminal inverted repeats (marked 1 and 2) of the IS to generate 3’OH ends (small green circles) that attack the target site (red arrows) in what is called a strand transfer reaction. DNA replication generates a cointegrate containing two IS copies in direct repeat together with a new target site duplication (white arrows). This structure can be subsequently resolved into a plasmid identical to the original donor plasmid and a modified target plasmid carrying an IS copy flanked by target site duplications arranged as direct repeats. **b:** replicative cointegration by an IS6-family pseudo-transposon [8] (modified from [1]. The symbols are identical to those above. The transposon is composed of two directly repeated copies of the IS flanking a DNA segment carrying passenger gene (green) with the internal flanks represented by yellow arrows. A target plasmid is distinguished by an open oval representing the origin of replication. Transposase-mediated replicon fusion of the two molecules using one of the two flanking IS copies generates a third copy of the IS in the same orientation as the original pair. Homologous recombination, using the *recA* system, between any two copies can in principle occur. This will either regenerate the donor plasmid, leaving a single IS copy in the target, delete the transposon, or transfer the transposon to the target (as shown), leaving a single copy of the IS in the donor molecule

Transposition of IS*6* family elements to generate cointegrates [12, 16, 18, 19] presumably occurs in a replicative manner. As shown in Fig. 5 (top), intermolecular replicative transposition of this type generates fused donor and target replicons which are separated by two copies of the IS in direct repeat at the replicon boundaries. The initial direct repeats (DR) flanking the donor IS are distributed between each daughter IS in the cointegrate as is the DR generated in the target site. Recombination between the two IS then regenerates the donor molecule with the original DRs and a target molecule in which the IS is flanked by new DR. No known specific resolvase system such as that found in Tn*3*-related elements has been identified in this family but “Resolution” of IS*6*-mediated cointegrates was observed to depend on a functional *recA* gene in several cases and therefore occurs using the host homologous recombination pathway [12, 16].

**Fig. 6:**
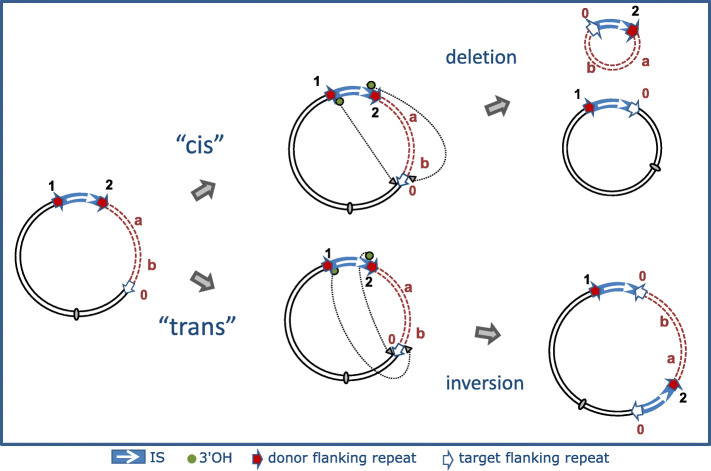
Intramolecular transposition. Symbols are identical to those in Fig. 5. The red dotted lines represent the DNA segment between the resident IS and its intramolecular target shown as a white arrow and marked “0”. In addition, a and b represent two markers on this DNA segment. The 3′-OH groups generated by cleavage at both IS ends can either attack the target site on the same strand (*cis*) (top pathway) or the opposite strand (*trans*) (bottom pathway). When *in cis*, DNA between the IS and target site is deleted as a circle containing the markers “a” and “b”, one IS copy flanked by one copy of the original flank, 2, and one copy of the target flank, 0. The other partner also contains a single IS copy with one copy of the original flank, 1, and one copy of the target flank, 0. When the reaction occurs *in trans*, DNA between IS and target site is instead inverted (“a b” becomes “b a”), bracketed by the original IS and a new copy in an inverted orientation. The target site is also duplicated but in inverted orientation, and one copy of the original flank and one copy of the target flank is associated with each IS copy

While the intermolecular cointegrate pathway leads to replicon fusion, transposition can also occur within the same replicon. Intramolecular transposition using the replicative mechanism gives rise to deletion or inversion of DNA located between the IS and its target site. The outcome depends on the orientation of the two attacking IS ends (Fig. 6). Intramolecular transposition of this type can explain the assembly of antibiotic resistance gene clusters (e.g. [76]).

IS*6* family members are known to generate structures that resemble composite transposons in which a passenger gene (such as a gene specifying antibiotic resistance) is flanked by two IS copies. Generally, the flanking IS in these compound structures can occur as direct or inverted repeat copies. However, in the case of IS*6* functional “compound transposons”, the flanking IS are always found as direct repeats. This is a direct consequence of the (homologous) recombination event required to resolve the cointegrate structure [12, 16]. As shown in Fig. 5 (bottom) [1], transposition is initiated by one of the flanking IS to generate a cointegrate structure with three IS copies. “Resolution” resulting in transfer of the transposon passenger gene requires recombination between the “new” IS copy and the copy which was not involved in generating the cointegrate. The implications of this model [1, 8] are that the transposon passenger gene(s) are simply transferred from donor to target molecules in the “resolution” event and are therefore lost from the donor “transposon”. Clearly this pathway could initiate from a donor in which the flanking IS*6* family members were inverted with respect to each other. However, transposition would be arrested at the cointegrate stage because there is no suitable second IS to participate in recombination. It is for this reason that compound IS*6*-based transposons carry directly repeated flanking IS copies. These previously published models (e.g. [1, 8, 76, 98] have recently been revisited and it has been recently proposed [23] that the term pseudo-compound transposons first used over 30 years ago [8] should be resurrected to describe these IS*6* family structures.

### Circular transposon molecules: translocatable units (TU)

**Fig. 7:**
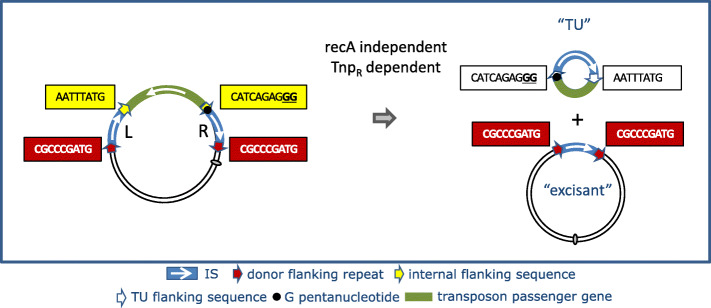
Summary of analysis of TU formation from the mutant transposon Tn4352B. The authors [106] used an IS26-based transposon, Tn*4352B*, carrying the *aphA1* gene in which the right hand IS fortuitously carried an additional GG dinucleotide at the left internal end of one of the component IS*26* copies to generate a GGGGG pentanucleotide at the IS tip. This appears to render the transposon unstable resulting in an excision of a non-replicative circle, called a translocatable element (TU), carrying a single IS copy and the *aphA1* gene. The other partner, the parental plasmid from which the TU had been excised, retained one IS copy and the original 8 base pair direct target repeat (framed in red). The sequence of the IS flanks in the TU were not reported. Symbols are the same as those used in Fig. 6

Although IS*26* transposition appears to be replicative with formation of cointegrate molecules, results from in vivo experiments suggest that its transposition may be more complex [107]. The idea that IS*26* might mobilize DNA in an unusual way arose from the observation that IS*6* family members can often be found in the form of arrays [106, 107] which could be interpreted as overlapping pseudo-compound transposons [23] (Figs. 4 and 5). Note that IS*26* and potential IS*26*-based transposons do not necessarily carry flanking direct target repeats but, as is the case for other TE which transpose by replicative transposition such as members of the Tn*3* family, intramolecular transposition would lead to loss of the flanking repeats (Fig. 6). This led to the suggestion that IS*26* might be able to transpose via a novel circular form called translocatable units (TU) [106, 107] (not to be confused with those originally described in the sea urchin and other eukaryotes [109]) such as those shown in Fig. 7. These potential circular transposition intermediates which were proposed to include a single IS*26* copy along with neighboring DNA are structurally similar to IS*1*-based circles observed in the 1970s (e.g. [85, 88]). Translocatable units differ from the transposon circles identified during copy-out-paste-in transposition by IS of the IS*3* (see [110]), IS*21* [111], IS*30* [112], IS*256* [113, 114] and IS*L3* [115] families where the circular IS transposition intermediate has abutted left and right ends separated by a few base pairs and is extremely reactive to the cognate transposase. In stark contrast, for IS*26*, the IS ends would be separated by the neighboring DNA sequence rather than by a few base pairs (Fig. 7).

Evidence for the excision step of translocatable units was obtained [106] from the study of the stability of two IS*26*-based pseudo-compound transposons, “wildtype” Tn*4352* [34] and “mutant” Tn*4352B* [116] which carry the *aphA1* gene specifying resistance to kanamycin. Tn*4352B* is a special mutant derivative of Tn*4352* including an additional GG dinucleotide at the left internal end of one of the component IS*26* copies to generate a string of 5 G nucleotides at the IS tip which appears to render the transposon unstable. Cells carrying the plasmid lose the resistance gene from the mutant Tn*4352B* at an appreciable rate in the absence of selection. This generates a “donor” plasmid with one copy of IS*26* flanked by the original Tn*4352B*-associated 8 bp direct repeats and an excision product with the size expected for a TU containing the second IS flanked by the sequences of the original central segment presumably including the additional GG dinucleotide together with the *aphA1* gene. TU formation, as judged by a PCR reaction, appeared to be dependent on the GG insertion (since surprisingly, no TU could be detected from the wildtype Tn*4352*) but not on the surrounding sequence environment. Excision required an active transposase. In a test in which the target plasmid also carried an IS*26* copy (a targeted integration reaction – see below), there appeared to be no difference in cointegrate formation frequencies between single IS*26* copies with or without the additional GG dinucleotide. However, results from a standard integration test into a plasmid without a resident IS*26* copy were not reported. The excision process occurs in a *recA*^*−*^ background and therefore does not require the host homologous recombination system. Moreover frameshift mutations in both IS, which should produce severely truncated transposase, eliminated activity. This implies that the process is dependent on transposition. However, excision continued to occur if the transposase of the GG-IS copy was inactivated but was eliminated when the same transposase mutation was introduced into the” wildtype” IS copy. This is curious since it implies that the IS*26* transposase must act exclusively *in cis* on the IS from which it is expressed.

A summary of these results is shown in Fig. 7. These data suggest that excision is driven by the wildtype IS*26* (L), leaving the right hand IS in the excisant. At present, there is no obvious mechanistic explanation for this phenomenon. It should be noted that recombination between directly repeated copies of IS*1* which flank the majority of antibiotic resistance genes in the plasmid R100.1 (NR1) generates a non-replicative circular molecule, the r-determinant (r-det), with a single IS*1* copy. In this case too, this “constitutive” circle production is due to a (uncharacterized) mutation in the plasmid, although in this case, circle production requires *recA* [117].

**Fig. 8:**
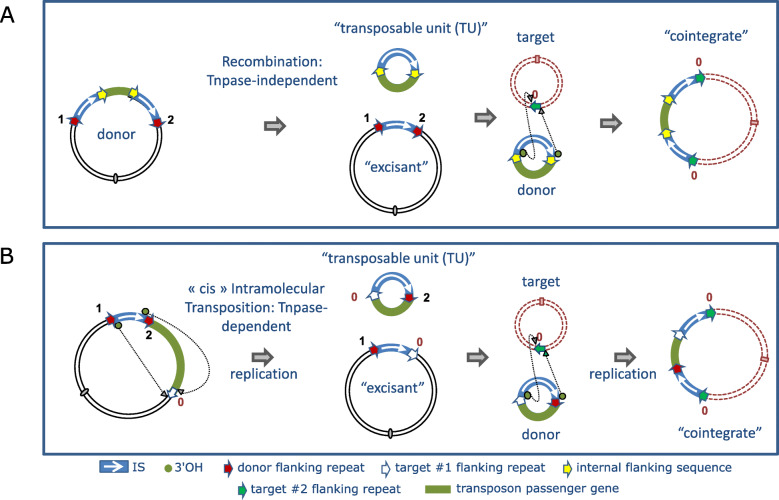
Two models for TU formation. Formally, both models would result in formation of a TU. **Top:** recA-dependent simple homologous recombination from a IS26-based pseudo-compound transposon leading to excision and transposase-dependent replicative transposition leads to a cointegrate. **Bottom:** Intramolecular transposition *in cis* from a donor with a single IS*26* leads to excision and transposase-dependent replicative transposition leads to a cointegrate

**Fig. 9:**
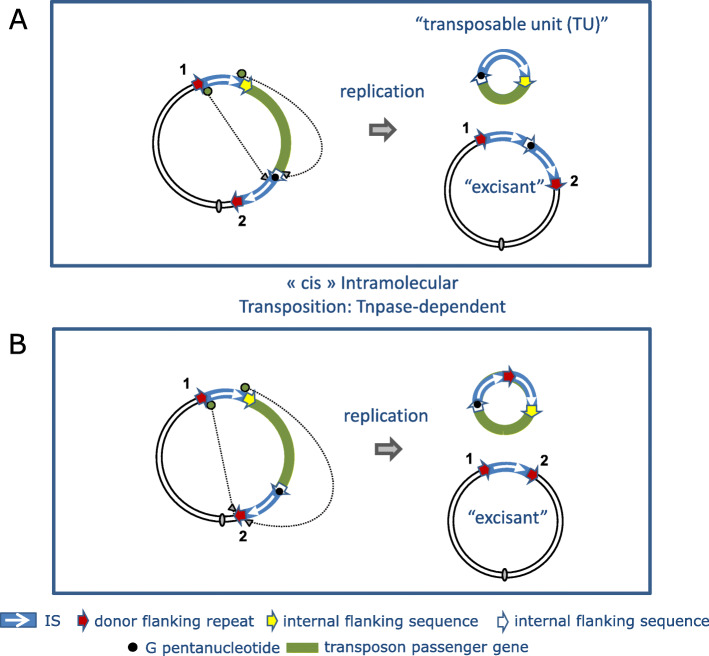
Two Models for TU formation from the Pseudo-compound Transposon Tn4325B. Symbols are as in the previous figures. The small filled circle within one of the internal IS flanks (white arrow) indicates the additional GG dinucleotide carried by Tn4325B. Both use an intramolecular replicative transposition pathway in a cis configuration. In the top panel, the wildtype IS uses the flank of the mutated IS as a target. This would generate a TU with a single IS and both internal flanking sequences and an excisant with two tandem IS copies separated by a mutant flank. In the lower panel, the TU carries two tandem IS copies and the excisant

However, “Classical” recombination and transposition models do not fit the data. The results appear to rule out two obvious models (Fig. 8) since although both would generate the correct TU and “excisant”, the first (Fig. 8 top panel) requires homologous recombination between two directly repeated IS*26* copies (mechanistically equivalent to the “resolution” step in intermolecular IS*6* transposition) and the second (Fig. 8 bottom panel), which requires a functional transposase as observed [106, 107], would not generate the correct flanking sequences. Modification of the transposition model to take into account the entire transposon (Fig. 9) in which the active IS*26*L uses either of flanking sequences of IS*26*R does not generate the correct structures. Thus the observed structures must be generated by another, and at present unknown, pathway. One possibility is that TU are generated by reversing a non-replicative targeted insertion mechanism presented below (see **Targeted Transposition**).

To summarize: it has been clearly demonstrated that circular DNA species carrying a single IS*26* copy together with flanking “passenger” DNA can be generated efficiently in vivo from a variant plasmid replicon [116] and also that replicons carrying a single IS*26* copy are capable of integrating into a second replicon to form a cointegrate. This occurs at a frequency 10^2^-fold higher if the target plasmid contains a single IS copy and in a targeted manner not involving IS duplication.

The TU insertion pathway was addressed by transforming TU, constructed in vitro taking advantage of a unique IS*26* restriction site, into recombination deficient cells carrying an appropriate target plasmid [105]. Establishment of the *aphA1*-carrying TU was dependent on the presence of a resident plasmid carrying an IS*26* copy and occurred next to the resident IS*26* copy. The DNA of two TU each with a different antibiotic resistance gene was shown to undergo this type of targeted integration and, moreover, were able to consecutively insert to generate a typical IS*26* array. Therefore, artificially produced TU are capable of insertion.

### Targeted transposition

**Fig. 10:**
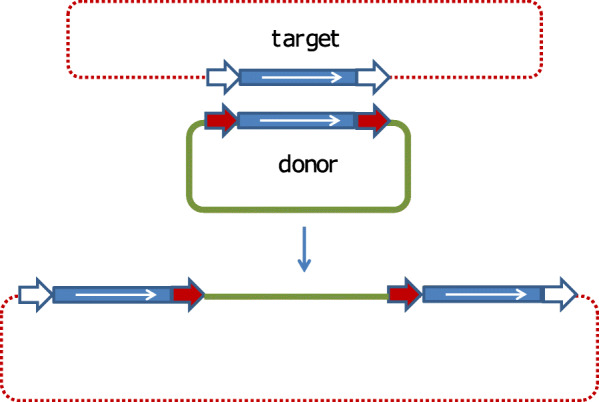
IS*26* Non-replicative Targeted Transposition. Symbols are identical to those in previous figures. The diagram shows the fate of flanking sequences following a targeted integration event resulting in the formation of a cointegrate

Targeted IS*26* transposition was also observed in intermolecular cointegrate formation where the cointegrate formation frequency was significantly increased (about 100 fold) if the target replicon also contained an IS*26* copy [107]. A similar result was obtained in *Escherichia coli* with a related IS, IS*1216* [118] whereas a third member of the family, IS*257* (IS*431*) showed a much lower level of activity using the same assay. As for TU integration, this phenomenon does not appear to be the result of homologous recombination between the IS copies carried by donor and target molecules since the reaction was independent of RecA. Using a PCR-based assay to identify the replicon fusions between IS*26*-containing donor and target plasmids, it was observed that the resulting cointegrate (Fig. 10) did not contain an additional copy of IS*26* which would be expected if replicative transposition were involved (Fig. 9). This suggests that the phenomenon results from a conservative recombination mechanism. Despite the absence of RecA, the observed cointegrate is structurally equivalent to the recombination product between the two IS*26* copies in the donor and target plasmids. However, it indeed appears to be transposition related since the phenomenon requires an active transposase in both donor and target replicons [107]. When each of the triad of conserved DDE residues were mutated individually in the donor plasmid, the targeted insertion frequency decreased significantly.

Another characteristic of the products was that the flanking 8 bp repeats carried by the donor and recipient IS*26* copies are in some way exchanged [107] (Fig. 10). This suggests a model in which transposase might catalyze an exchange of flanking DNA during the fusion process.

### A model for targeted integration

**Fig. 11:**
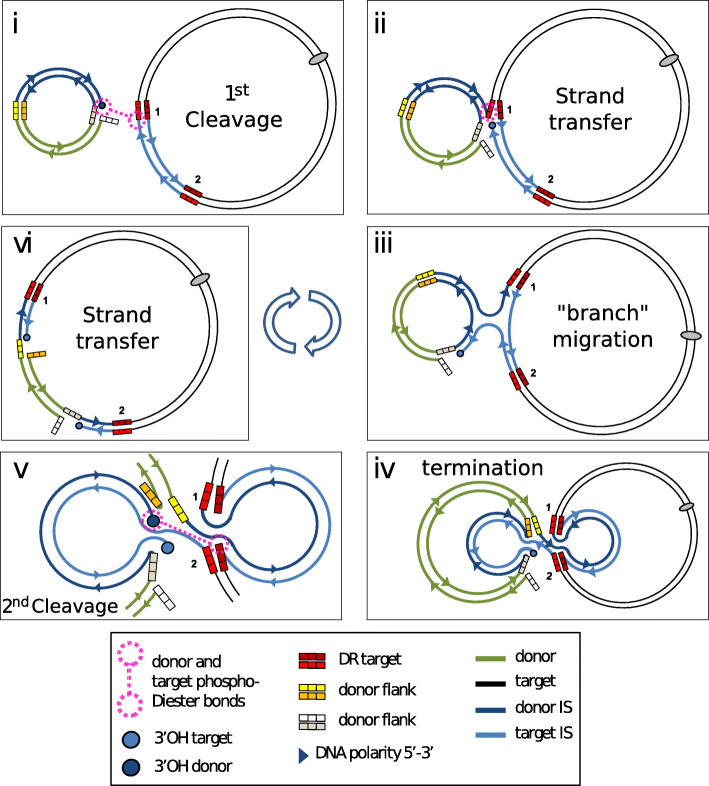
A model for IS26-mediated conservative targeted integration. **i)** Two IS ends from different IS copies in separate replicons are synapsed intermolecularly in the same transpososome, one end is cleaved to generate a 3’OH (shown as a dark blue circle) leaving a 5′ and on the flank (3 white boxes). This attacks the end of the second IS in the transpososome (shown as two dotted circles joined by a dotted line). **ii)** strand transfer would then couple the donor and target replicons via the target IS flank (3 bright red squares) leaving a 3’OH on the target IS (light blue circle). **iii)** strand migration can then occur in which one strand of the door IS and one strand of the target IS invade their partners. **iv)** following exchange of the entire partner strands, only a single physical strand cleavage would have occurred leaving a single single-strand break (three white squares). **v)** a second strand cleavage at the distal end of the donor IS occurs (dark blue circle) leaving its free 5′ flank (three orange squares). The 3’OH then attacks the distal target IS end (shown as two dotted circles joined by a dotted line). **vi)** strand transfer then generates a cointegrate with single-strand nicks at each end on opposite strands (white and orange squares) which could then be repaired. Note that the cointegrate retains the original flanking repeats of the target IS (three bright red and three dark red squares)

One possibility (Fig. 11) is that two IS ends from different IS copies in separate replicons are synapsed intermolecularly in the same transpososome (Fig. 11i). Strand exchange would then couple the donor and target replicons (Fig. 11 ii). A similar mechanism has been invoked to explain “targeted” insertion of IS*3* and *IS30* family members into TIRs [119, 120]. Branch migration (Fig. 11 iii) would lead to exchange of an entire IS strand (Fig. 11 iv) and cleavage at the distal IS end and strand transfer (Fig. 11v) would result in the observed cointegrate (Fig. 11 vi) containing a single strand nick on opposite strands at each end of the donor DNA molecule. These could be repaired or eliminated by plasmid replication. Each IS would be composed of complementary DNA strands from each of the original donor and target IS copies. This proposed mechanism would retain the DNA flanks of the IS in the original target replicon, be dependent on an active transposase and independent of the host recA system. It seems probable that mismatches between the two participant IS would inhibit the strand migration reaction. This may be the reason for the observation that introducing a frameshift mutation by insertion of additional bases into the transposase gene of either participating IS*26* copy reduces the frequency of targeted cointegration [107] since, not only does this produce a truncated transposase but also introduces a mismatch. As in the case of intermolecular targeting of the IS*3* family member, IS*911* [121], might require the RecG helicase to promote strand migration.

The model shown in Fig. 11 presents the transposition process as a progression involving two consecutive temporally separated strand cleavages separated by a strand migration. However, it seems equally probable that both cleavage reactions are coordinated within a single transpososome including both donor IS ends and the target IS ends. This would be compatible with the known properties of *trans* cleavage of several transposases in which a transposase molecule bound to one transposon end catalyzes cleavage of the opposite end. Recently, evidence has been presented supporting this type of model [122]. Using two IS, IS*1006* and IS*1008* [123] which have significant identity to IS*26* their ends, together with a hybrid molecule IS*1006*/*1008* constructed in vitro, it was shown that targeted integration required both identical transposases and identical DNA sequences at the reacting ends. The authors propose a model in which a single IS end is cleaved and transferred to the flank of the target IS end, an event which creates a Holliday junction which, on branch migration, is resolved. This differs from the model shown here (Fig. 11) since it does not involve transposase-mediated cleavage at the second IS end. It is similar to that proposed for targeted insertion of IS*911* [119, 121, 124, 125] which requires the RecG helicase and, presumably, RuvC.

## Conclusions and future directions

We have presented a survey of our present knowledge concerning the properties, distribution and activities of IS*6* family members and their importance, in particular that of IS*26*, in gene acquisition and gene flow of antibacterial resistance in enterobacteria. There are many questions which remain to be answered and we feel that some care should be excersied in interpreting some of the very interesting results in the absence of formal proof. For example, the notion that the basic IS*6* family transposition unit is a non-replicative circular DNA molecule carrying a single IS copy is attractive and would provide a nice parallel to the integron antibiotic resistance gene cassette intermediates [126–128] but such a molecule, a TU, has thus far been formally observed in only a single case. It was generated in vivo from an IS*26*-flanked peudo-transposon in which one of the two flanking IS involved included a mutation and rendered the transposon unstable. The “wildtype” transposon was stable [106]. Since “TU” is now being used in the literature to describe IS*26*-flanked DNA segments in multimeric arrays (e.g. [93], it is essential to provide more formal evidence that these non-replicative DNA circles are indeed general intermediates in the IS*26* transposition pathway and are not simply amplified units (AU). The fact that a replicating plasmid containing a single IS copy is able to form cointegrates does not a priori support a model for TU transposition and is not necessarily simply a TU that has the capacity to replicate [107] although the observation that artificially constructed TU can undergo targeted insertion when introduced into a suitable cell by transformation [105] supports the TU hypothesis. A second important question to be answered is how targeted integration occurs. We have suggested one model and suggested ways it might be tested (Fig. 11). The answers to many of these fascinating outstanding questions will be only be formally provided when the biochemistry of the reactions is known.

## Supplementary Information

**Additional file 1: Figure S1a-g.** Left (IRL) and right IRR and a combined IRL + IRR inverted terminal repeats for each clade are shown in WebLogo format [25]. **Figure S1h.** The last section shows an alignment of the ends of clade Aiv adjusted by hand.

**Additional file 2: Figure S2.** Alignment of a representative sample of the transposases of IS6 family members including members of each major clade. Alignment was by Clustal [129] and the graphic output from SnapGene. The figure shows the probable zinc finger N-terminal extension (consecutive CxxC motifs), the helix-turn-helix domain (HTH) and the catalytic domain (DDE K/R).

## Data Availability

The data are available from the corresponding author and from ISfinder (https://www-is.biotoul.fr/), TnCentral (https://tncentral.proteininformationresource.org/) and TnPedia (https://tnpedia.fcav.unesp.br/).
