# Supplementary material for: The IS6 family, a clinically important group of insertion sequences including IS26
Source: Mob DNA. 2021 Mar 23;12:11. doi: 10.1186/s13100-021-00239-x (PMC7986276; doi:10.1186/s13100-021-00239-x)
Supplement: Supplementary file 1 — Additional file 1: Figure S1a-g. Left (IRL) and right IRR and a combined IRL + IRR inverted terminal repeats for each clade are shown in WebLogo format [25]. Figure S1h. The last section shows an alignment of the ends of clade Aiv adjusted by hand. [file 13100_2021_239_MOESM1_ESM.pdf]

Fig. S1 a

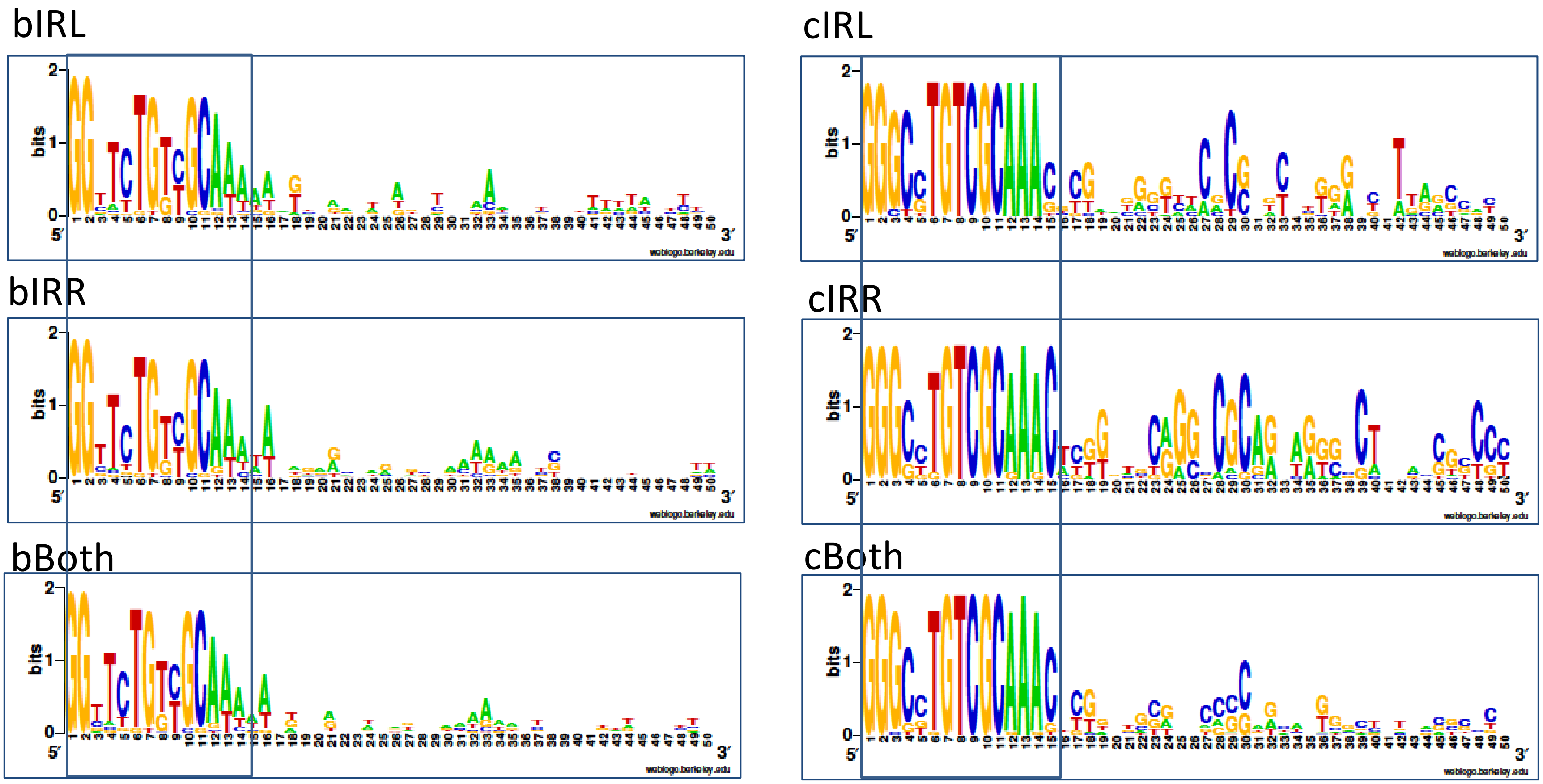
bIRL
cIRL
bIRR
cIRR
bBoth
cBoth
bits
5′
3′
weblogo.berkeley.edu

Fig. S1 b

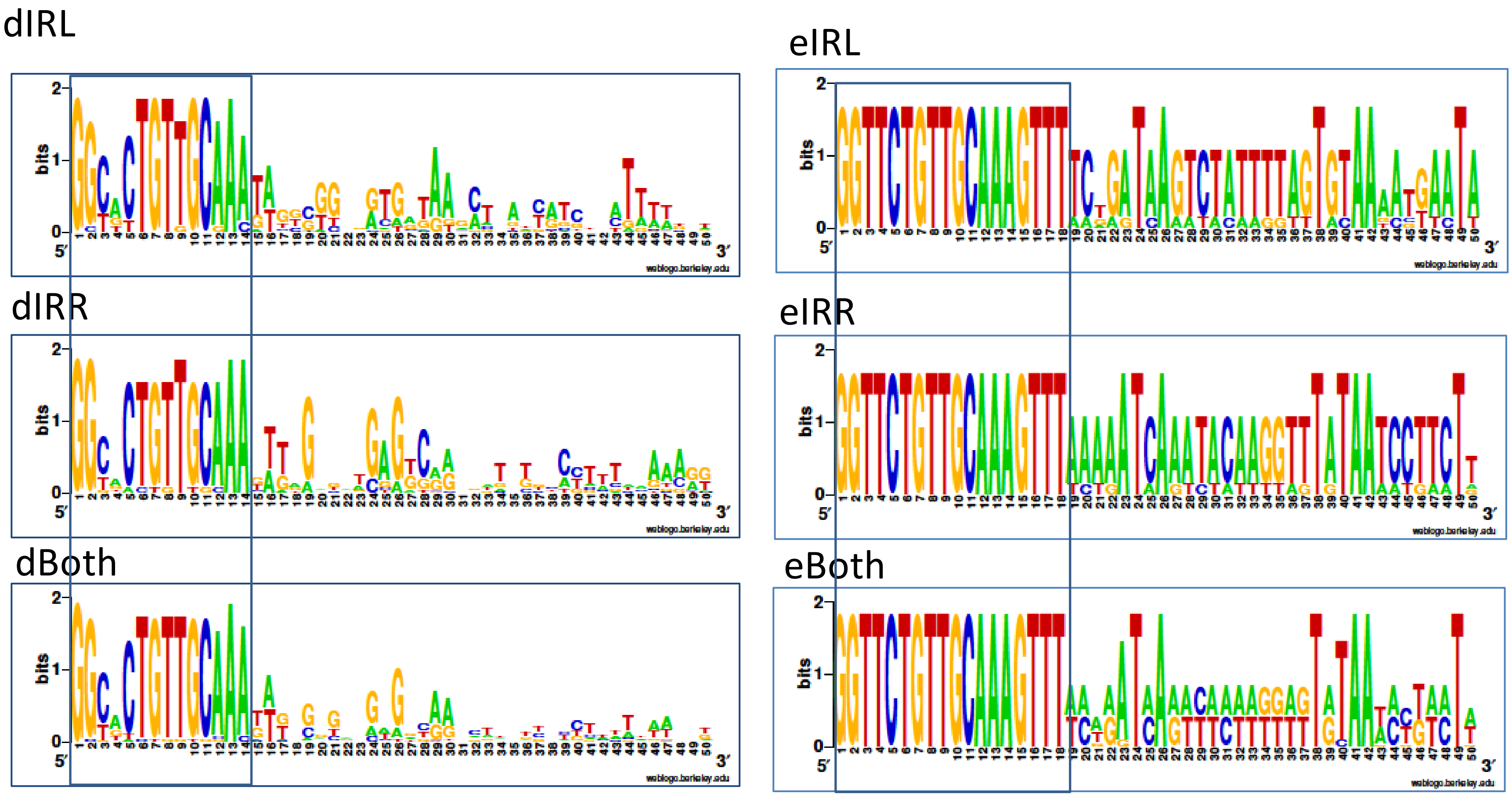

Fig. S1 c

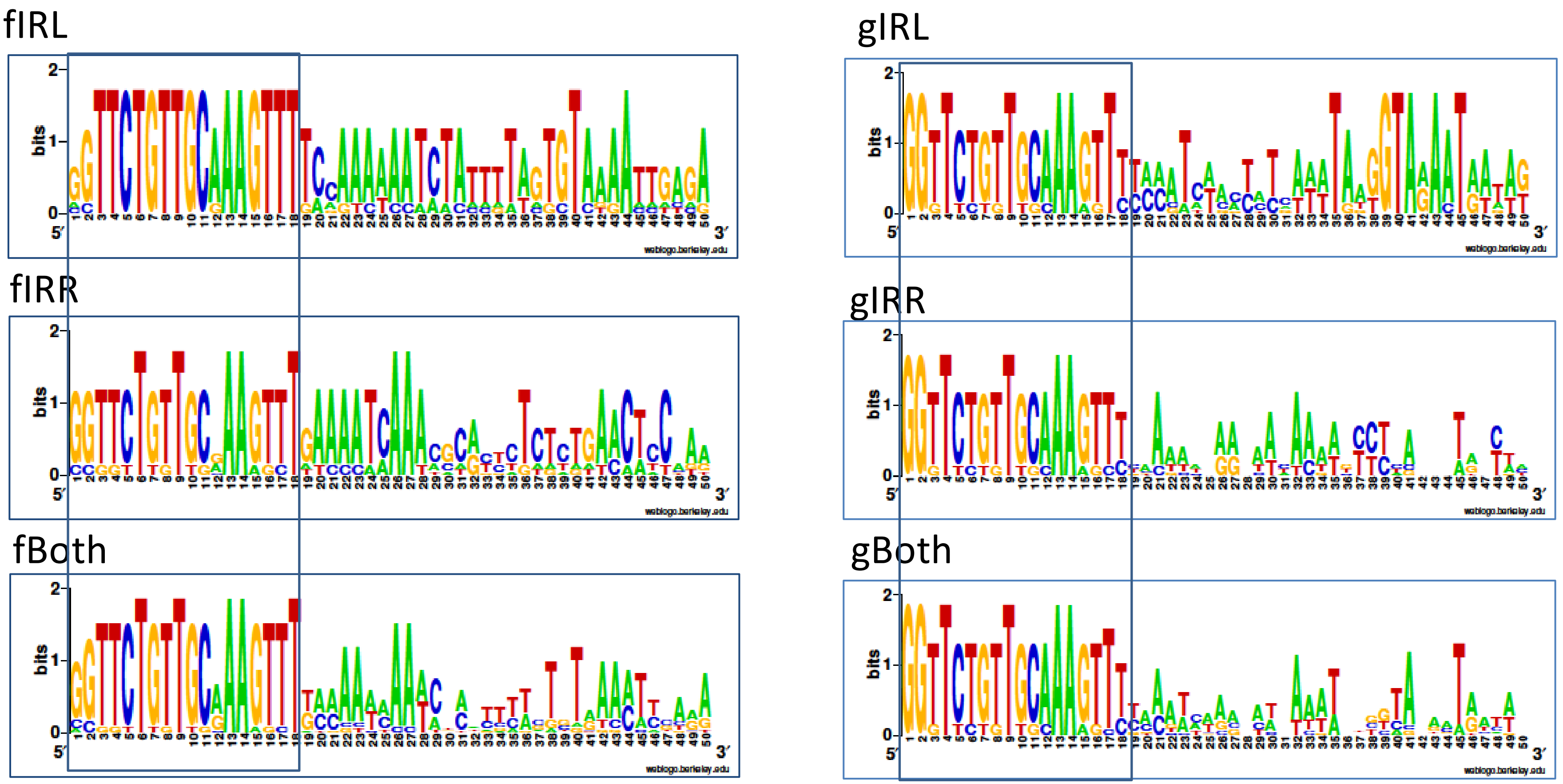
fIRL
gIRL
fIRR
gIRR
fBoth
gBoth
bits
5′
3′
weblogo.berkeley.edu

Fig. S1 d

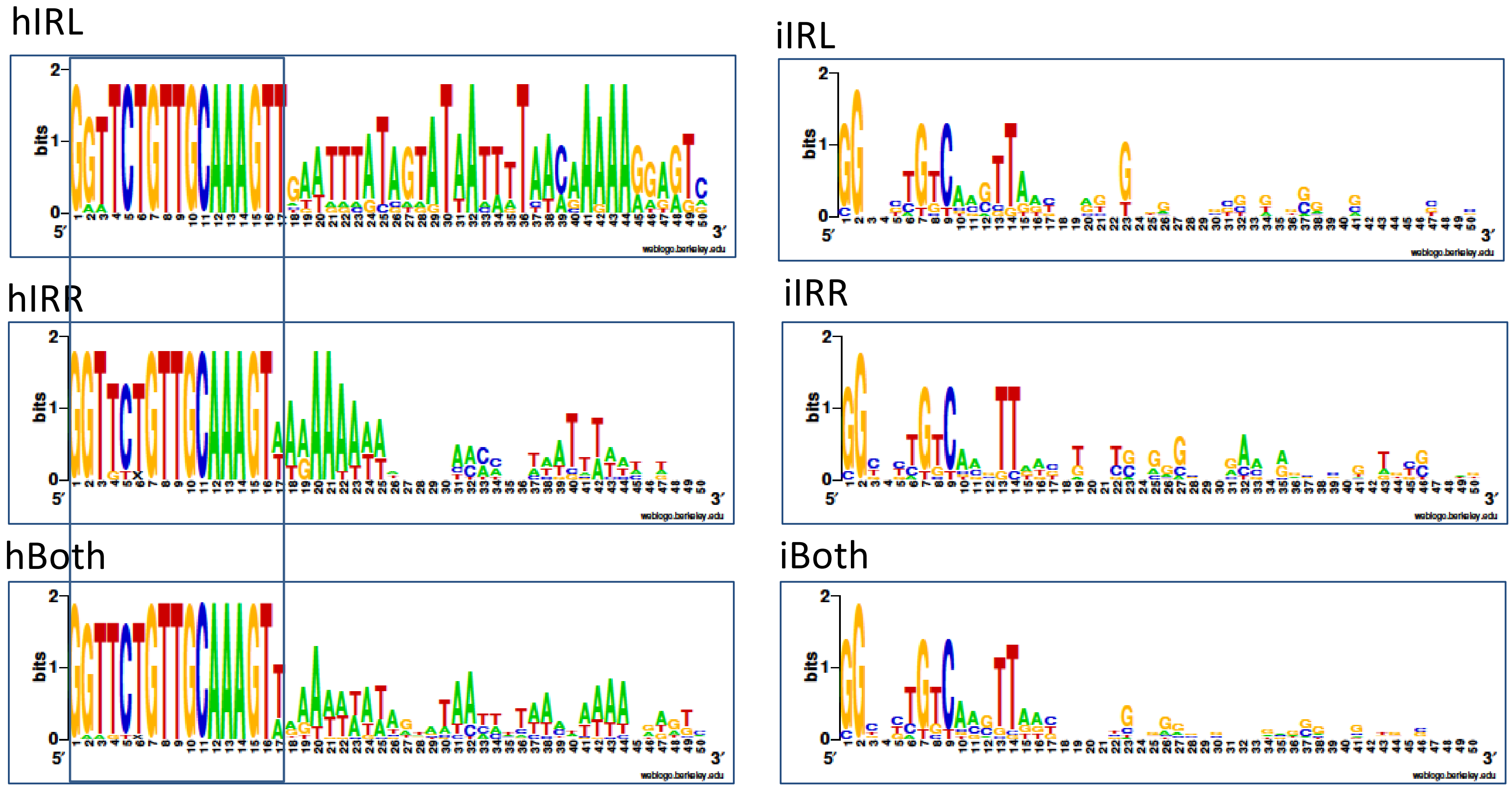
hIRL
iIRL
hIRR
iIRR
hBoth
iBoth
bits
5′
3′
weblogo.berkeley.edu

Fig. S1 e

jIRL

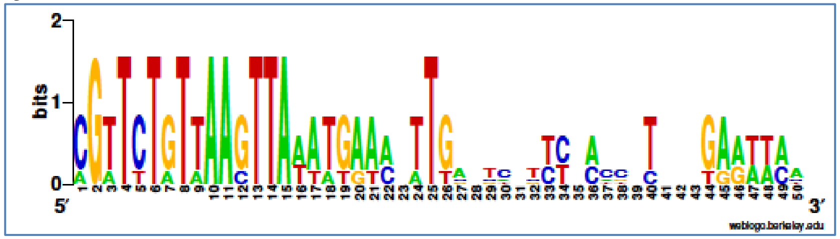

jIRR

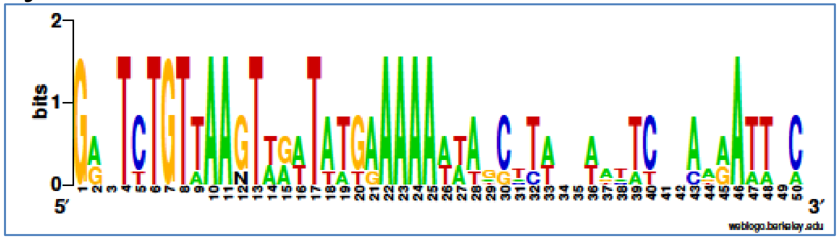

jBoth

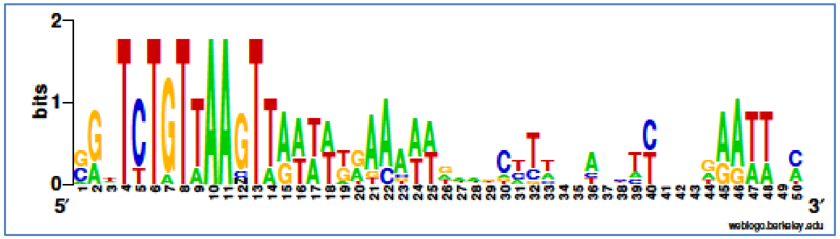

Fig. S1 f

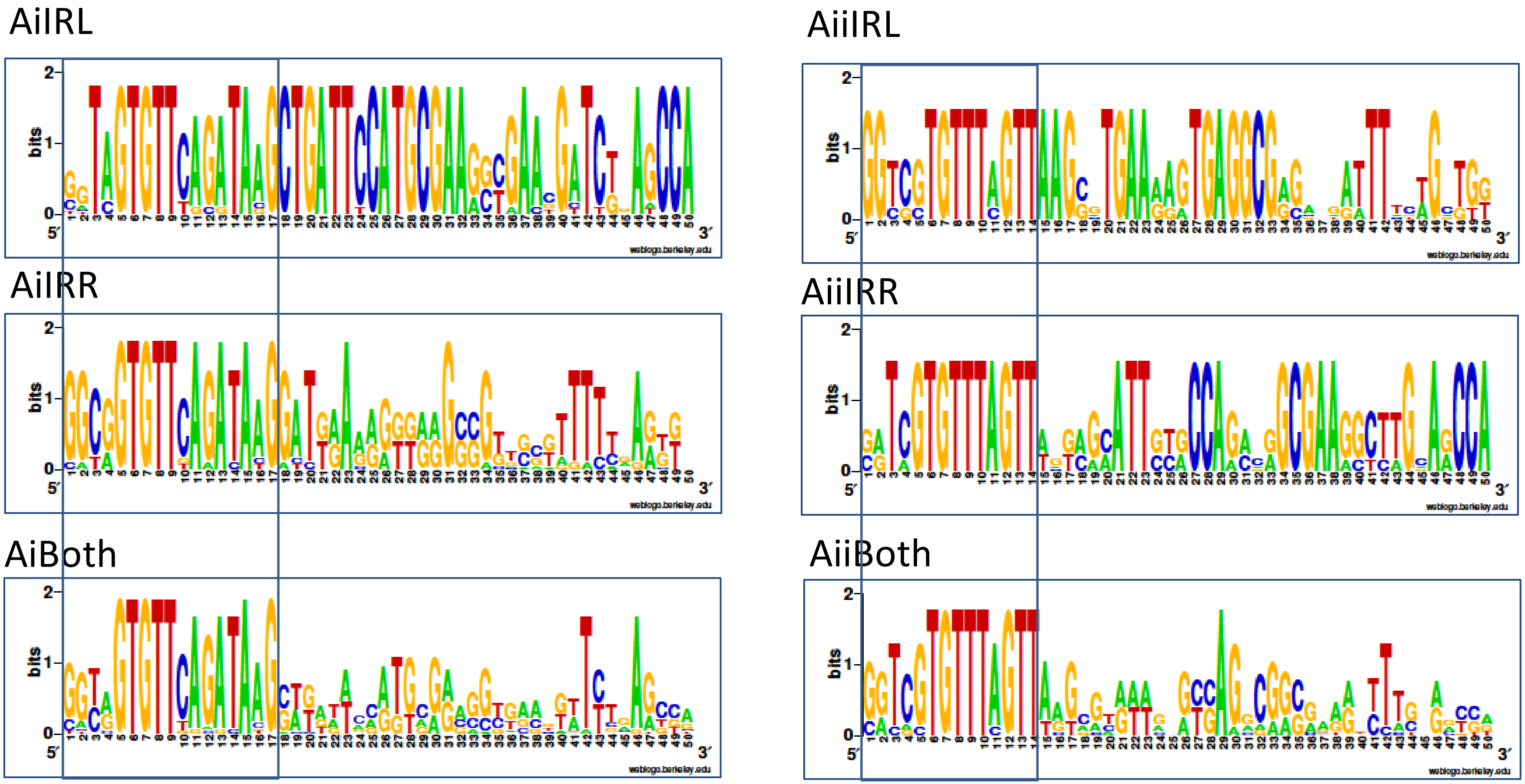
AiIRL
AiiIRL
AiIRR
AiiIRR
AiBoth
AiiBoth
bits
5′
3′
weblogo.berkeley.edu

Fig. S1 g

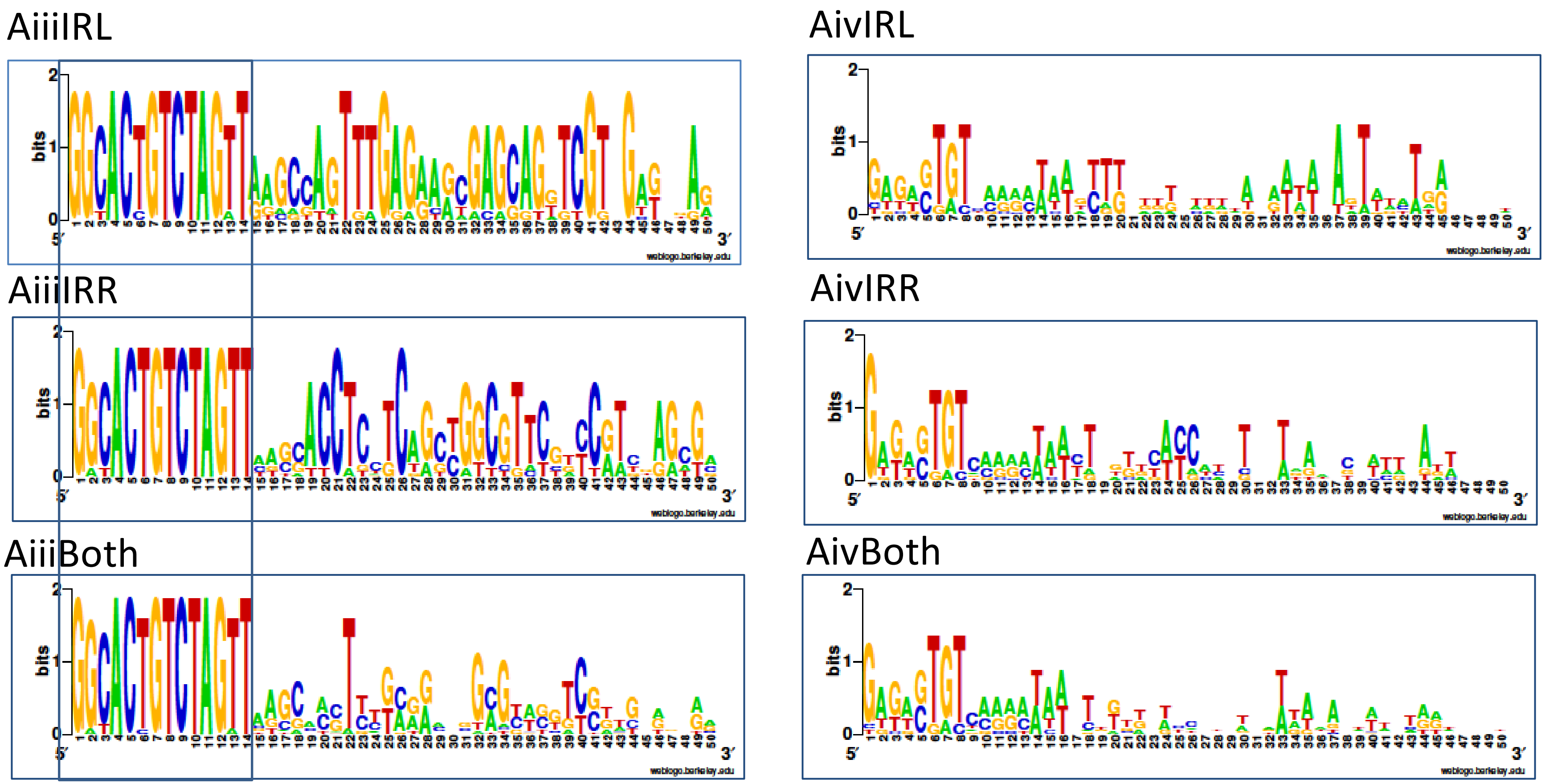

Fig. S1 h

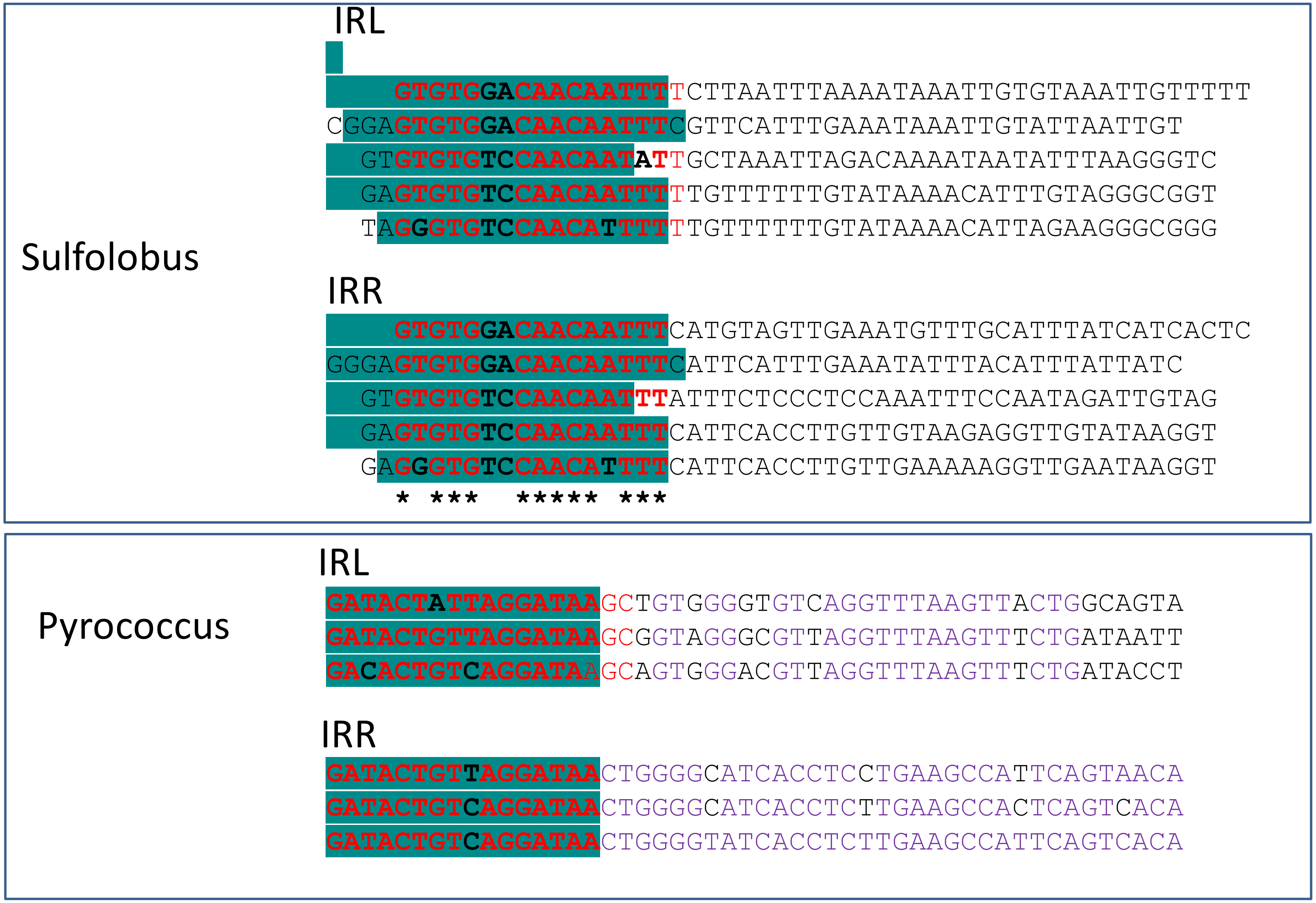

Sulfolobus
IRL
GTGTGGACAACAATTTTCTTAATTTAAAATAAATTGTGTAAATTGTTTTT
CGGAGTGTGGACAACAATTTCGTTCATTTGAAATAAATTGTATTAATTGT
GTGTGTGTCCAACAATATTGCTAAATTAGACAAAATAATATTTAAGGGTC
GAGTGTGTCCAACAATTTTTGTTTTTTGTATAAAACATTTGTAGGGCGGT
TAGGGTGTCCAACATTTTTTGTTTTTTGTATAAAACATTAGAAGGGCGGG
IRR
GTGTGGACAACAATTTCATGTAGTTGAAATGTTTGCATTTATCATCACTC
GGGAGTGTGGACAACAATTTCATTCATTTGAAATATTTACATTTATTATC
GTGTGTGTCCAACAATTTATTTCTCCCTCCAAATTTCCAATAGATTGTAG
GAGTGTGTCCAACAATTTCATTCACCTTGTTGTAAGAGGTTGTATAAGGT
GAGGGTGTCCAACATTTTCATTCACCTTGTTGAAAAAGGTTGAATAAGGT
Pyrococcus
IRL
GATACTATTAGGATAAGCTGTGGGGTGTCAGGTTTAAGTTACTGGCAGTA
GATACTGTTAGGATAAGCGGTAGGGCGTTAGGTTTAAGTTTCTGATAATT
GACACTGTCAGGATAAGCAGTGGGACGTTAGGTTTAAGTTTCTGATACCT
IRR
GATACTGTTAGGATAACTGGGGCATCACCTCCTGAAGCCATTCAGTAACA
GATACTGTCAGGATAACTGGGGCATCACCTCTTGAAGCCACTCAGTCACA
GATACTGTCAGGATAACTGGGGTATCACCTCTTGAAGCCATTCAGTCACA
