# Supplementary figures and images for: The IS6 family, a clinically important group of insertion sequences including IS26
Source: Mob DNA. 2021 Mar 23;12:11. doi: 10.1186/s13100-021-00239-x (PMC7986276; doi:10.1186/s13100-021-00239-x)

Fig. S2

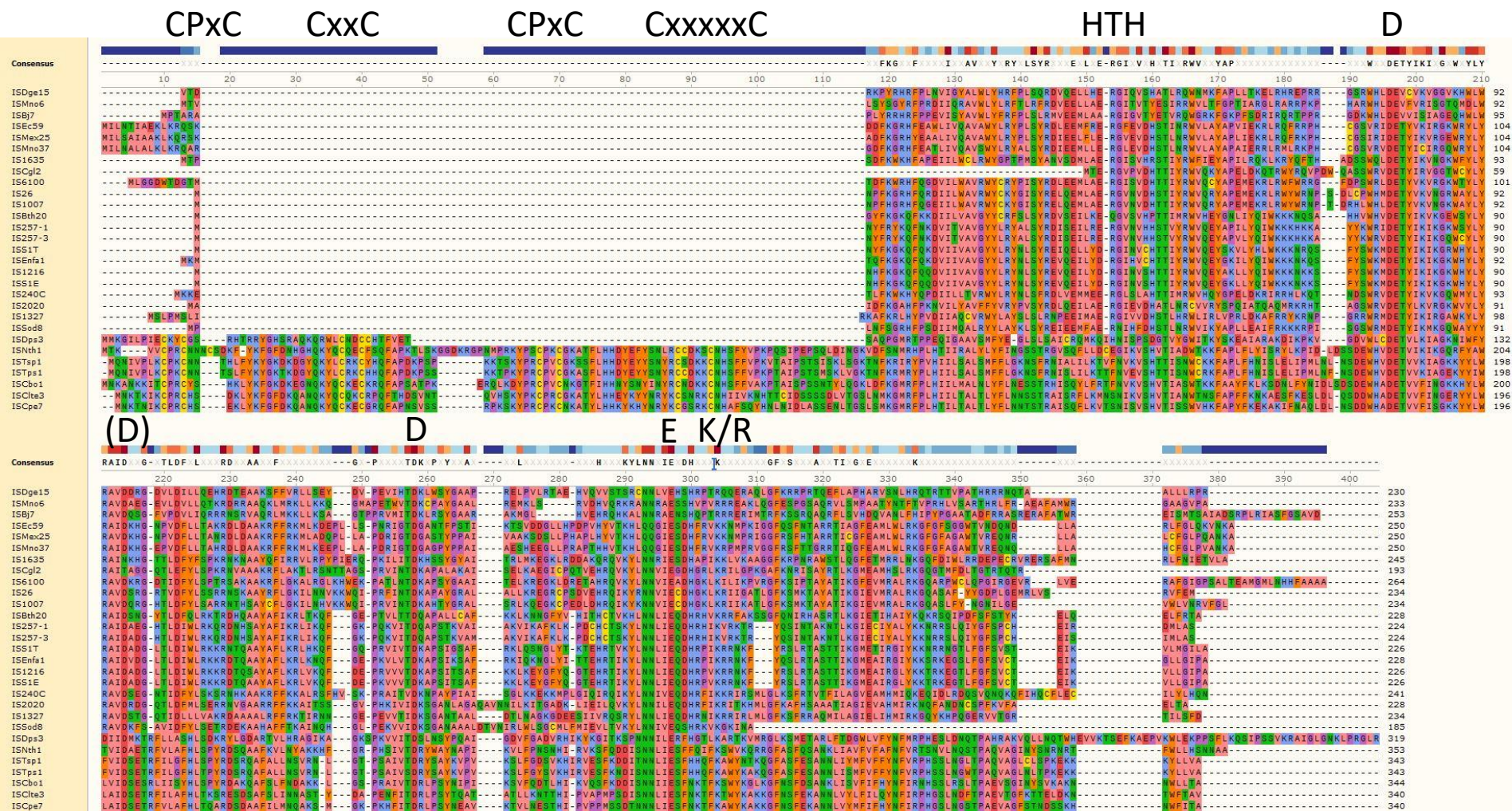

Supplement: Supplementary file 2 — Additional file 2: Figure S2. Alignment of a representative sample of the transposases of IS6 family members including members of each major clade. Alignment was by Clustal [129] and the graphic output from SnapGene. The figure shows the probable zinc finger N-terminal extension (consecutive CxxC motifs), the helix-turn-helix domain (HTH) and the catalytic domain (DDE K/R). [file 13100_2021_239_MOESM2_ESM.pdf]
